# The Chloride Homeostasis of CA3 Hippocampal Neurons Is Not Altered in Fully Symptomatic *Mepc2-null* Mice

**DOI:** 10.3389/fncel.2021.724976

**Published:** 2021-09-17

**Authors:** Yasmine Belaïdouni, Diabe Diabira, Jinwei Zhang, Jean-Charles Graziano, Francesca Bader, Aurelie Montheil, Clément Menuet, Gary A. Wayman, Jean-Luc Gaiarsa

**Affiliations:** ^1^Aix-Marseille University UMR 1249, Institut de Neurobiologie de la Méditerranée, Institut National de la Santé et de la Recherche Médicale Unité 1249, Parc Scientifique de Luminy, Marseille, France; ^2^Institute of Biomedical and Clinical Sciences, College of Medicine and Health, University of Exeter, Hatherly Laboratories, Exeter, United Kingdom; ^3^Aix-Marseille University 105, Institut Paoli Calmettes, U1068, Institut National de la Santé et de la Recherche Médicale U7258, Centre National de Recherche Scientifique, Centre de Recherche en Cancérologie de Marseille, Marseille, France; ^4^Program in Neuroscience, Department of Integrative Physiology and Neuroscience, Washington State University, Pullman, WA, United States

**Keywords:** KCC2, NKCC1, Rett, GABA, hippocampus

## Abstract

Rett syndrome (RTT) is an X-linked neurodevelopmental disorder caused mainly by mutations in the *MECP2* gene. Mouse models of RTT show reduced expression of the cation-chloride cotransporter KCC2 and altered chloride homeostasis at presymptomatic stages. However, whether these alterations persist to late symptomatic stages has not been studied. Here we assess KCC2 and NKCC1 expressions and chloride homeostasis in the hippocampus of early [postnatal (P) day 30–35] and late (P50–60) symptomatic male *Mecp2-null (Mecp2*^–/*y*)^ mice. We found (i) no difference in the relative amount, but an over-phosphorylation, of KCC2 and NKCC1 between wild-type (WT) and *Mecp2*^–/*y*^ hippocampi and (ii) no difference in the inhibitory strength, nor reversal potential, of GABA_*A*_-receptor-mediated responses in *Mecp2*^–/*y*^ CA3 pyramidal neurons compared to WT at any stages studied. Altogether, these data indicate the presence of a functional chloride extrusion mechanism in *Mecp2*^–/*y*^ CA3 pyramidal neurons at symptomatic stages.

## Introduction

Rett syndrome (RTT) is a non-inherited chromosome X-linked neurodevelopmental disorder characterized by loss of acquired speech, hand stereotypies, and gait abnormalities which appear following a period of apparently normal postnatal development. Other frequent symptoms include breathing difficulties, seizures, scoliosis, and growth retardation (Neul et al., 2010). RTT accounts for up to 10% of severe intellectual disability of genetic origin in women. Typical RTT cases arise from *de novo* loss-of-function mutations in the *MECP2* gene encoding the Methyl-CpG-binding protein 2 (MECP2) (Amir et al., 1999), a global transcriptional regulator. Given the location of the *MECP2* gene on the X chromosome, males with *MECP2* mutations are more severely affected and rarely survive infancy, while females, owing to X chromosome inactivation, are mosaics with cells that express either the wild-type (WT) or mutant version of *MECP2* (Chahrour and Zoghbi, 2007). Several clinical features of the human disorder are recapitulated in *Mecp2*-deficient mice (Chen et al., 2001; Guy et al., 2001; Shahbazian and Zoghbi, 2002) and therefore represent a crucial tool for deciphering the cellular mechanisms of the disease and for testing potential treatment strategies (Katz et al., 2012).

The K^+^-Cl^–^ co-transporter 2 (KCC2), responsible for the outward transport of chloride, is one of the many factors deregulated in the context of RTT. Both mRNA and protein KCC2 expressions are reduced in postmortem hippocampus (Hinz et al., 2019), motor cortex, and cerebellum (Gogliotti et al., 2018), as well as in the cerebrospinal fluid (Duarte et al., 2013) of RTT patients. The expression of the Na^+^-K^+^-Cl^–^ co-transporter 1 (NKCC1), which is responsible for the inward transport of chloride, is not affected, leading to a reduced KCC2/NKCC1 ratio (Gogliotti et al., 2018; Hinz et al., 2019) and likely to intracellular chloride accumulation in RTT brains. Along the same line, KCC2 expression is reduced and chloride homeostasis is altered in human neurons differentiated from RTT patients (Tang et al., 2016, 2019). A decrease of KCC2 expression and impaired chloride homeostasis was also reported in the visual cortex (Banerjee et al., 2016; Scaramuzza et al., 2021) and hippocampus (El-Khoury et al., 2014; Lozovaya et al., 2019) of *Mecp2*-null mice. However, most of these investigations were performed at stages when the symptoms are not overt (Banerjee et al., 2016; Lozovaya et al., 2019; Scaramuzza et al., 2021), and whether these alterations persist to late symptomatic stages has been recently questioned (Oyarzabal et al., 2020).

Here, we investigated the expression of the chloride co-transporters NKCC1 and KCC2, as well as the chloride homeostasis in the hippocampus of symptomatic male *Mecp2*-null mice. Western blot and qRT-PCR analysis revealed no differences in the total expression of KCC2 and NKCC1 at the transcriptional and translational levels. Post-translational (i.e., phosphorylation) differences were, however, observed. Whole cell and perforated patch clamp revealed no difference in the inhibitory strength, nor reversal potential, of GABA_*A*_-receptor-mediated responses in *Mecp2*^–/*y*^ CA3 pyramidal neurons compared to WT at any stages studied.

## Materials and Methods

### Animals

All animal procedures were carried out in accordance with the European Union Directive of 22 September (2010/63/EU) and have been approved by the Ethical Committee for Animal Experimentation (APAFIS-Number 17605-2018-1119-1115-7094) delivered by the French Ministry of Education and Research. Experiments were performed on male WT and *Mecp*^2*tm*1–1*Bird*^ mice (Guy et al., 2001) C57BL/6 genetic background (Jackson Laboratory, stock number 003890). Hemizygous mutant males (*Mecp2*^–/*y*^) were generated by crossing heterozygous females (*Mecp2*^±^) with C57BL/6 WT males. Animals were housed in a temperature-controlled environment with a 12 h light/dark cycle and free access to food and water. Genotyping was performed by PCR techniques according to Jackson Laboratory protocols.

### Hippocampal Slice Preparation

Brains were removed and immersed into ice-cold (2–4°C) artificial cerebrospinal fluid (ACSF) with the following composition: 126 mM NaCl, 3.5 mM KCl, 2 mM CaCl_2_, 1.3 mM MgCl_2_, 1.2 mM NaH_2_PO_4_, 25 mM NaHCO_3_, and 11 mM glucose (pH 7.4) equilibrated with 95% O_2_ and 5% CO_2_. Hippocampal slices (350 μm thick) were cut using a vibrating microtome (Leica VT1000S, Germany) in ice-cold oxygenated choline-replaced ACSF and were allowed to recover at least 90 min in ACSF at room temperature (25°C). Slices were then transferred to a submerged recording chamber perfused with oxygenated (95% O_2_ and 5% CO_2_) ACSF (3 ml/min) at 34°C.

### Electrophysiological Recordings

*Perforated patch-clamp recordings* were made from CA3 pyramidal neurons. The pipettes (4–7 MΩ) were tip filled with an internal solution of 150 mM KCl and 10 mM HEPES (pH adjusted to 7.2 with Tris-OH) and then backfilled with the same solution containing gramicidin A (50 μg/ml). Data were acquired with an axopatch 200B amplifier (Molecular Devices LLC, San Jose, CA, United States). A stimulating bipolar tungsten electrode was placed in the CA3 *stratum radiatum* to evoke GABA_*A*_-receptor-mediated postsynaptic currents (eGABA_*A*_-PSCs) at a frequency of 0.01 Hz in the presence of glutamatergic receptor antagonists (NBQX 5 μM and D-APV 40 μM). The intensity of stimulation was usually set to evoke the maximum amplitude response. After the access resistance had dropped to 40–80 MΩ and stabilized (15–30 min), we varied the starting holding potential (–70 mV) in increasing and decreasing steps of 10 mV and measured the peak amplitude of averaged eGABA_*A*_-PSCs (three single sweeps) to construct a current–voltage relationship. A linear regression was used to calculate the best-fit line of the voltage dependence of the synaptic currents. Spontaneous rupture into whole-cell was evidenced by large inward synaptic currents due to E_*Cl*_ of 0 mV.

*Whole cell recordings* were performed from CA3 pyramidal neurons in the voltage-clamp mode using an axopatch 200B amplifier (Molecular Devices LLC, San Jose, CA, United States). To assess the excitatory/inhibitory balance, the glass recording electrodes (4–7 MΩ) were filled with a solution containing 100 mM K-gluconate, 13 mM KCl, 10 mM Hepes, 1.1 mM EGTA, 0.1 mM CaCl_2_, 4 mM Mg–adenosine 5′-triphosphate, and 0.3 mM Na–guanosine 5′-triphosphate. The pH of the intracellular solution was adjusted to 7.2, and the osmolality was adjusted to 280 mOsmol/l. With this solution, GABA_*A*_-PSCs reversed at –70 mV. GABA_*A*_-PSCs and Glut-PSCs were recorded at a holding potential of –45 and –70 mV, respectively. To assess the rate of chloride extrusion, the pipettes were filled with the above solution supplemented with 17 mM KCl (final concentration 30 mM) and eGABA_*A*_-PSCs were evoked at various holding potential at a frequency of 0.01 Hz in the presence of NBQX 5 μM and D-APV 40 μM. The specific KCC2 blocker, VU0463271, was applied at a concentration of 10 μM during 20 min, to assess the presence of functional KCC2 transporter. A linear regression was used to calculate the best-fit line of the voltage dependence of the synaptic currents.

*Loose cell attached patch-clamp recordings* of action potential firing were performed from CA3 pyramidal neurons in the voltage-clamp mode at a pipette potential of 0 mV using an axopatch 200B amplifier (Molecular Devices LLC, San Jose, CA, United States). The glass electrodes (4–7 MΩ) were filled with an internal solution of 150 mM KCl and 10 mM HEPES (pH adjusted to 7.2 with Tris-OH). After a baseline period of at least 10 min, isoguvacine (10 μM) was bath applied for 2 min.

*Extracellular multiunit activity recordings (MUA)* were performed from CA3 pyramidal neurons using extracellular tungsten 50 μm electrodes (California Fine Wire) which were placed in the pyramidal cell layer. Data were acquired using a DAM80i amplifier (WPI, Sarasota, FL, United States). After a baseline period of at least 10 min, isoguvacine (10 μM) was bath applied for 2 min.

Evoked synaptic activity, spontaneous synaptic currents, action potentials, and MUA were recorded with Axoscope software version 8.1 (Molecular Devices LLC, San Jose, CA, United States) and analyzed offline with Mini Analysis Program version 6.0 (Synaptosoft).

### Real-Time Reverse Transcription Quantitative Polymerase Chain Reaction

Mice were sacrificed, and hippocampi were dissected and rapidly frozen in liquid nitrogen and stored at –80°C. RNAs were isolated and quantified by reading the absorbance at 260 nm (NanoPhotometer, IMPLEN) using Mini RNeasy kit (Qiagen), and then converted to cDNA using 1 mg RNA and a QuantiTect Reverse Transcription kit (Qiagen) according to the manufacturer’s instructions. PCR was carried out with the LightCycler 480 SYBR Green IMaster (Roche Applied Science) with 1 mL cDNA using the following oligonucleotides (QuantiTectPrimer Assays, Qiagen): NKCC1 (Slc12a2; QT00197785), KCC2 (Slc12a5; QT00145327), and glyceral-dehyde-3-phosphate dehydrogenase (GAPDH, QT001199633). Quantitative RT-PCR was performed with a Roche LC480 Light Cycler (Roche Applied Science) following the manufacturer’s instructions. Relative mRNA values were calculated using the LC480 software and GAPDH as the housekeeping gene. PCR was performed in replicate of three.

### Western Blot and Immunoprecipitation With Phosphorylation Site-Specific Antibodies

Mice were sacrificed, and hippocampi were dissected and rapidly frozen in liquid nitrogen and stored at –80°C until protein extraction. Hippocampi were lysed in lysis buffer [50 mM Tris/HCl, pH 7.5, 1 mM EGTA, 1 mM EDTA, 50 mM sodium fluoride, 5 mM sodium pyrophosphate, 1 mM sodium orthovanadate, 1% (w/v) Triton-100, 0.27 M sucrose, 0.1% (v/v) 2-mercaptoethanol, and protease inhibitors (complete protease inhibitor cocktail tablets, Roche, one tablet per 50 ml)], and protein concentrations were determined following centrifugation of the lysate at 16,000 × *g* at 4°C for 20 min using the Bradford method with bovine serum albumin as the standard. Tissue lysates (15 μg) in SDS sample buffer (1X NuPAGE LDS sample buffer (Invitrogen) containing 1% (v/v) 2-mercaptoethanol) were subjected to electrophoresis on polyacrylamide gels and transferred to nitrocellulose membranes. The membranes were incubated for 30 min with TBS-Tween buffer [TTBS, Tris/HCl, pH 7.5, 0.15 M NaCl, and 0.2% (v/v) Tween-20] containing 5% (w/v) skim milk. The membranes were then immunoblotted in 5% (w/v) skim milk in TBS with the indicated primary antibodies overnight at 4°C. The blots were then washed six times with TBS and incubated for 1 h at room temperature with secondary HRP-conjugated antibodies diluted 5,000-fold in 5% (w/v) skim milk in TTBS. After repeating the washing steps, the signal was detected with the enhanced chemiluminescence reagent. Immunoblots were developed using ChemiDoc^TM^ Imaging Systems (Bio-Rad). Primary antibodies used were: anti-KCC2 phospho-Ser940 (Thermo Fisher Scientific, cat #PA5-95678), anti(neuronal)-β-Tubulin III (Sigma-Aldrich, cat #T8578), anti-KCC2 total (University of Dundee, S700C), anti-phospho-Thr906 (University of Dundee, S959C), anti-KCC2 phospho-Thr1007 (University of Dundee, S961C), anti-NKCC1 total antibody (University of Dundee, S022D), and anti-NKCC1 phospho-Thr203/Thr207/Thr212 antibody (University of Dundee, S763B). Horseradish peroxidase-coupled (HRP) secondary antibodies used for immunoblotting were from Pierce. The relative intensities of immunoblot bands were determined by densitometry with ImageJ software.

### Plethysmography

The breathing activity of freely moving 7–8-week-old male mice was recorded using a constant flow whole body plethysmograph (EMKA technologies, Paris, France) with 200 ml animal chambers maintained at 25 ± 0.5°C and ventilated with air (600 ml/min). The breathing activity of pairs of WT and *Mecp2*^–/*y*^ mice littermates was simultaneously recorded. Mice were allowed to acclimate to the experimental room for 1 h and to the plethysmography chamber and airflow for approximatively 30 min before breathing measurement. Breathing activity was recorded during 1 h. A differential pressure transducer measured the changes in pressure in the body chamber resulting from the animal’s respiration. Signals from the pressure transducer were stored on a personal computer and analyzed offline via the Spike 2 interface and software (Cambridge Electronic Design, Cambridge, United Kingdom). Only periods of quiet breathing without body movements were analyzed, during which the number of apneas (>two respiratory cycles) per hour was quantified.

### Accelerating Rotarod

A rotarod apparatus (Biological Research Apparatus, Gemonio, Italy) was used to measure the motor coordination. After a 10-min habituation session (4 rpm), each mouse was given three trials with the rate of rotation increasing from 4 to 40 rpm over 5 min. The trial ended when the mouse fell from the rod or remained on the rotarod for at least 5 min. The time spent on the rotarod was recorded by an automated unit, which stopped as the mouse fell. The mouse was placed back in its home cage for 10 min between each trial. The latency to fall was determined as the average of the three trials.

### Drugs

The following reagents were purchased from the indicated sources: 1,2,3,4-Tetrahydro-6-nitro-2,3-dioxo-benzo [f]quinoxaline-7-sulfonamide (NBQX) and D-2-amino-5-phospho-valeric acid (D-APV) from the Molecular, Cellular, and Genomic Neuroscience Research Branch (MCGNRB) of the National Institute of Mental Health (NIMH, Bethesda, MD, United States) and Isoguvacine and VU0463271 from Tocris Cookson (Bristol, United Kingdom).

### Statistics

Statistical analyses were conducted with GraphPad Prism (GraphPad software 5.01). Shapiro–Wilk normality test was used to determine the normality of distributions. We used a two-tailed Mann–Whitney *U* test or two-tailed unpaired *t*-test for comparison between two independent groups. To ensure the consistency and reproducibility of our results, we conducted repeated trials in different acute hippocampal slices prepared from at least three different animals from three different littermates. All data are expressed as mean ± standard error of the mean (SEM). In the figures, box plots represent the first and third quartiles; whiskers show data range; and horizontal lines show the median.

## Results

**FIGURE 1:**
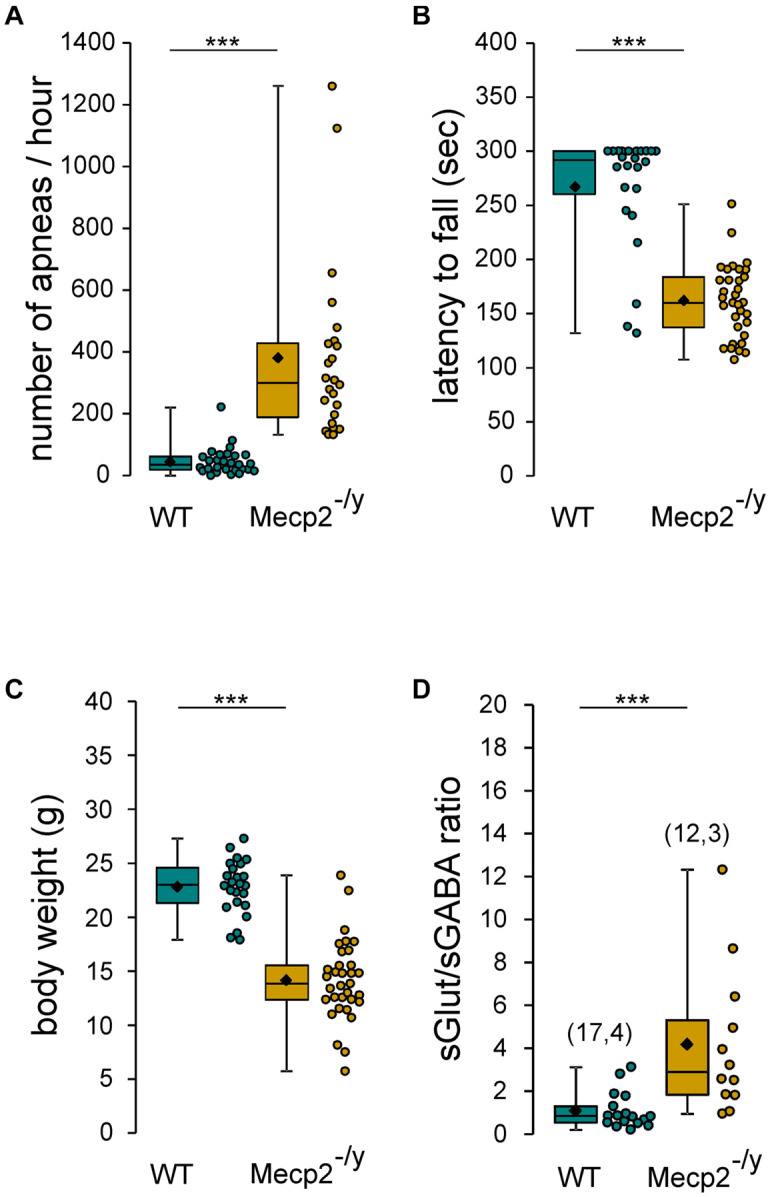
RTT features of *Mecp2*^–^ *^/y^* mice. Box plots of the number of apneas **(A)**, latency to fall from rotarod **(B)**, body weight **(C)**, and sGlut/sGABA frequency ratio **(D)** of 7–8 weeks-old wild-type (WT) and *Mecp2*^– /y^ mice. In this and following figures, box plots represent quartiles, whiskers show data range, lozenges represent arithmetic means, scatter plots show individual data points, and numbers in parentheses indicate the number of neurons/slices recorded and the number of mice used. ***P < 0.001, two-tailed Mann–Whitney *U* test.

The aim of this study was to assess the chloride homeostasis in the hippocampus of symptomatic *Mecp2*^–/*y*^ mice. We first ensure that the 6–7-week-old *Mecp2*^–/*y*^ mice of our breeding colony exhibit the RTT-like symptoms thoroughly reported in previous studies (Guy et al., 2001; Pratte et al., 2011; Calfa et al., 2015; Lozovaya et al., 2019), including (Figure 1) breathing abnormalities (*n* = 29 WT and 24 *Mecp2*^–/*y*^ mice, *U* = 7, *P* < 0.0001), motor dysfunction (*n* = 24 WT and 33 *Mecp2*^–/*y*^ mice, *U* = 71, *P* < 0.0001), body weight loss (*n* = 24 WT and 33 *Mecp2*^–/*y*^ mice, *U* = 29, *P* < 0.0001), premature death [22% of the *Mecp2*^–/*y*^ died before P50 (*n* = 18 out of 81 mice)], and increased synaptic excitatory/inhibitory balance onto CA3 pyramidal neurons (*n* = 17 and 12 neurons from, respectively, three WT and four *Mecp2*^–/*y*^ mice, *U* = 20, *P* = 0.0003).

### Post-translational Modifications of K^+^-Cl^–^ Co-transporter 2 and Na^+^-K^+^-Cl^–^ Co-transporter 1 in the Hippocampi of Symptomatic *Mecp2*^–/*y*^ Mice

**FIGURE 2:**
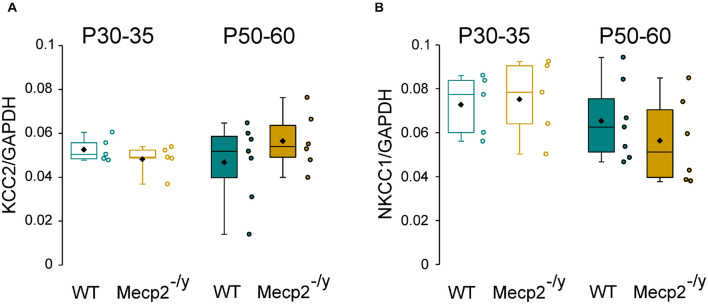
mRNA expression of KCC2 and NKCC1 in wild-type (WT) and *Mecp2*^– /y^ hippocampi. Box plots of normalized KCC2 **(A)** and NKCC1 **(B)** mRNA expression in the hippocampi of P30–35 and P50–60 WT and *Mecp2*^–^ *^/y^* mice.

**FIGURE 3:**
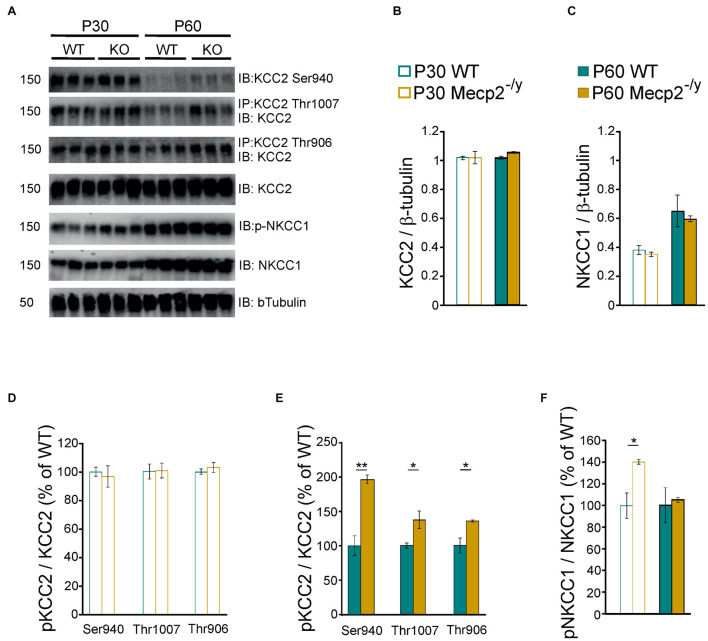
Protein expression of KCC2 and NKCC1 in wild-type and *Mecp2*^–^ *^/y^* hippocampi. Western blots **(A)** and quantifications of KCC2 **(B)**, NKCC1 **(C)**, and the phosphorylated forms of KCC2 **(D,E)** and NKCC1 **(F)** in P30–35 and P50–60 WT and *Mecp2*^–^ *^/y^* hippocampi. *n* = 3 for both. Mean ± SEM. *P < 0.05, **P < 0.01, two-tailed unpaired Student’s *t*-test.

Chloride homeostasis is mainly controlled by the activity of two cation-chloride cotransporters: NKCC1 that accumulates Cl^–^ intracellularly and KCC2 that lowers intracellular Cl^–^ concentration (Medina et al., 2014). We therefore probed mRNA and protein expressions of these two Cl^–^ co-transporters in WT and *Mecp2*^–/*y*^ littermates using quantitative RT-qPCR and western blot from isolated hippocampi. Both early (P30–35) and late (P50–60) symptomatic mice were analyzed. There was no difference in the relative amount of KCC2 and NKCC1 mRNA between WT and *Mecp2*^–/*y*^ mice at P30–35 (*U* = 10, *P* = 0.67, *n* = 5 for both) nor at P50–60 (*U* = 16, *P* = 0.35 and *U* = 14, *P* = 0.52, *n* = 7 WT and 6 *Mecp2*^–/*y*^ hippocampi) (Figure 2). The amount of KCC2 and NKCC1 proteins was also not different between the two genotypes at any of the postnatal stages tested (*P* = 0.7 at P30, *P* = 0.2 at P60, *n* = 3 for both; Figures 3A–C).

The transport activity of KCC2 and NKCC1 strongly depends on the (de)phosphorylation of several residues within their c-terminus domain (Medina et al., 2014; Shekarabi et al., 2017). Phosphorylation of threonine residues 906 and 1007 (KCC2-T906/T1007) decreases KCC2 activity, while phosphorylation of the serine 940 residue (KCC2-S940) enhances KCC2 activity. Phosphorylation of the threonine residues 203, 207, and 212 (NKCC1-T203/207/212) increases NKCC1 activity. We therefore assessed the phosphorylated state of KCC2 and NKCC1 in WT and *Mecp2*^–/*y*^ hippocampi. The normalized (to total KCC2) expressions of phospho-KCC2-S940, phospho-KCC2-T906, and phospho-KCC2-T1007 were not different between WT and *Mecp2*^–/*y*^ littermates at P30 (*P* = 0.7, 0.9, and 0.5, respectively), but was increased in *Mecp2*^–/*y*^ mice at P60 (*P* = 0.003, 0.04, and 0.03, respectively, *n* = 3 for both, Figures 3A,D,E). The normalized (to total NKCC1) expression of phospho-NKCC1-T203/207/212 was increased in *Mecp2*^–/*y*^ mice compared to WT littermates at P30 (*P* = 0.02), but not at P60 (*P* = 0.7, n = 3 for both, Figures 3A,F).

### The Strength of GABAergic Inhibition Onto CA3 Pyramidal Neurons Is Not Affected in Symptomatic *Mecp2*^–/*y*^ Mice

**FIGURE 4:**
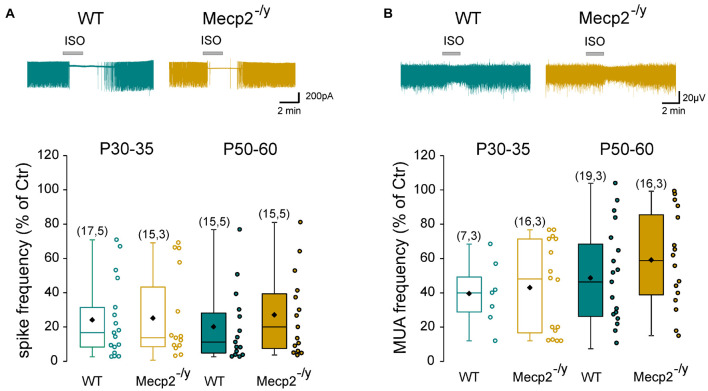
Strength of GABAergic inhibition onto wild-type and *Mecp2*^–^ *^/y^* CA3 hippocampal pyramidal neurons. Top traces: loose patch recordings **(A)** and field recordings **(B)** of CA3 pyramidal neurons on acute hippocampal slice from a P50 WT and *Mecp2*^–^ *^/y^* mouse. Box plots of isoguvacine effects normalized to the baseline spike frequency **(A)** or multi-unit activity (MUA) **(B)** in P30–35 and P50–60 WT and *Mecp2*^–^ *^/y^* mice.

Next, we determined whether the inhibitory strength of GABA was altered in *Mecp2*^–/*y*^ hippocampi. Loose patch-clamp recordings were made to test the effect of bath applied isoguvacine, a GABA_*A*_-receptor agonist (ISO, 10 μM), on the firing of CA3 pyramidal neurons. Isoguvacine transiently reduced ongoing spike frequency with the same efficacy in WT and *Mecp2*^–/*y*^ CA3 pyramidal neurons at P30–35 [24 ± 22% (*n* = 17) versus 25 ± 25% (*n* = 15) of control, *U* = 122, *P* = 0.89] and P50–60 (20 ± 21% versus 27 ± 24% of control, *n* = 15 for both, *U* = 88, *P* = 0.31, Figure 4A). Similar results were obtained with extracellular field potential recordings at P30–35 [39.5 + 18.9% (*n* = 7) versus 42.9 + 26.8% (*n* = 16) of control, *U* = 50, *P* = 0.71] and P50–60 [48.5 + 29% (*n* = 19) versus 59.1 + 28.1% (*n* = 16) of control, *U* = 117, *P* = 0.25, Figure 4B].

**FIGURE 5:**
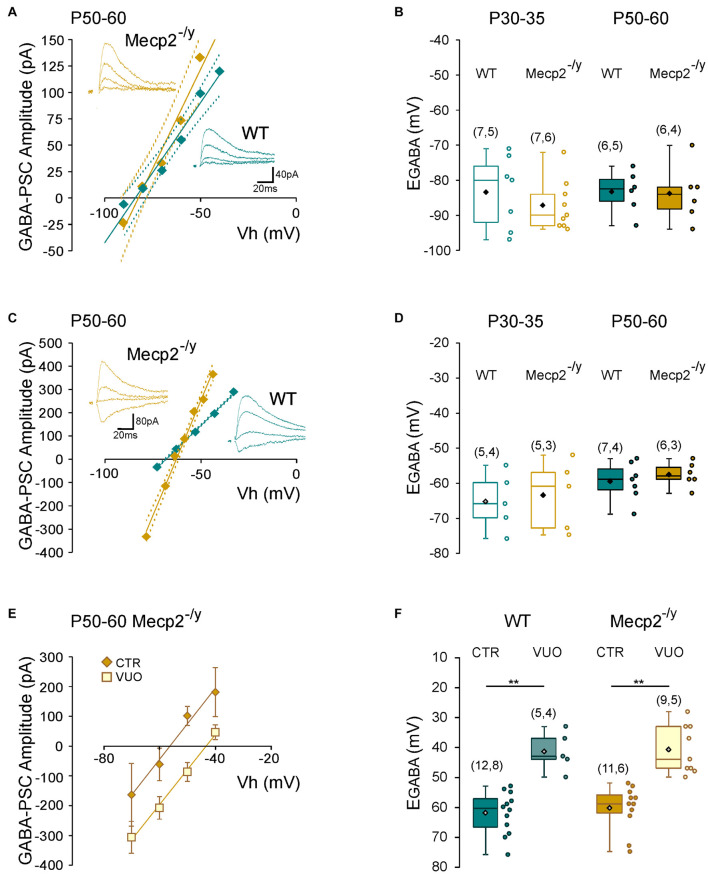
Chloride homeostasis onto wild-type and *Mecp2*^–^ *^/y^* CA3 hippocampal pyramidal neurons. Current–voltage relationships (solid lines) and confidence interval (95%, dotted lines) **(A,C)** and box plots **(B,D)** of the reversal potential of evoked GABAergic synaptic currents (E_*GABA*_) in WT and Mecp2^– /y^ CA3 pyramidal neurons. **(A,B)** Perforated gramicidin patch-clamp recordings. **(C,D)** Whole cell patch-clamp recordings. Insets: examples of GABAergic synaptic-currents evoked onto a P50–60 WT and *Mecp2*^–^ *^/y^* CA3 pyramidal neuron. **(E)** Current–voltage relationships of evoked GABAergic synaptic currents in P50–60 *Mecp2*^– /y^ CA3 pyramidal neurons and **(F)** E_*GABA*_ in WT and Mecp2^– /y^ CA3 pyramidal neurons in control condition (Ctr) and in the presence of VU0463271 (VUO,10 μM). **P < 0.01, two-tailed Mann–Whitney *U* test.

To directly probe possible alterations in chloride homeostasis in *Mecp2*^–/*y*^ CA3 pyramidal neurons, we measured the reversal potential of evoked GABA_*A*_-receptor-mediated postsynaptic current (E_*GABA*_) using gramicidin-perforated patch-clamp recordings. We found no significant difference in E_*GABA*_ between the two genotypes at any of the postnatal stages tested (*U* = 21.5, *P* = 0.3 at P30–35 and *U* = 16, *P* = 0.8 at P50–60, Figures 5A,B and Table 1). The other IPSCs’ parameters (amplitude, kinetic, and coefficient of variation of amplitude) as well as the estimated resting membrane potential (membrane potential at zero current) were also not different between WT and *Mecp2*^–/*y*^ CA3 pyramidal neurons at both stages (Table 1).

**TABLE 1:** Reversal potential of evoked GABA_*A*_-receptor-mediated synaptic current on WT and *Mecp2-null* CA3 hippocampal pyramidal neurons (two-tailed Mann–Whitney *U* test).

	P30–35			P50–60		
	WT	*Mecp2* ^–/*y*^	P	WT	*Mecp2* ^–/*y*^	P
eGABA_*A*_-PSCs amplitude (mV)	33 ± 7 (*n* = 7)	47 ± 10 (*n* = 7)	0.3	35 ± 9 (*n* = 6)	39 ± 12 (*n* = 6)	0.8
Coefficient of variation of eGABA_*A*_-PSCs amplitude	0.21 ± 0.05	0.14 ± 0.04	0.9	0.11 ± 0.03	0.066 ± 0.005	0.5
eGABA_*A*_-PSCs rise (ms)	4.2 ± 0.9	3.4 ± 0.7	0.8	5.2 ± 1.6	6.3 ± 2.1	0.8
eGABA_*A*_-PSCs decay (ms)	25 ± 3	22 ± 1	0.1	20 ± 8	17 ± 4	0.8
E_*GABA*_ (mV) (Perforated patch clamp)	–83.4 ± 3.9 (*n* = 7)	–84.4 ± 3.3 (*n* = 7)	0.7	–83.1 ± 2.3 (*n* = 6)	–83.8 ± 3.3 (*n* = 6)	0.8
Em (mV) (Perforated patch clamp)	–60.7 ± 2.3 (*n* = 7)	–59.1 ± 4.7 (*n* = 7)	0.9	–58.6 ± 1.2 (*n* = 6)	–57.1 ± 3.1 (*n* = 6)	0.6
E_*GABA*_ (mV) (Whole cell, chloride loading)	–65.4 ± 3.6 (*n* = 5)	–63.6 ± 3.3 (*n* = 5)	0.8	–59.5 ± 2.1 (*n* = 7)	–57.6 ± 1.4 (*n* = 6)	0.6

We also compared the rate of chloride extrusion between WT and *Mecp2*^–/*y*^ CA3 pyramidal neurons using a whole-cell patch-clamp chloride loading assay. By imposing a 30 mM chloride load through the recording pipette, according to the Nernst equation, E_*GABA*_ should equal −37 mV in the absence of chloride transport. WT and *Mecp2*^–/*y*^ CA3 pyramidal neurons displayed statistically equivalent E_*GABA*_ values at P30–35 (*U* = 11, *P* = 0.8) and P50–60 (*U* = 17, *P* = 0.6 at P50–60, Figures 5C,D and Table 1) that were more negative than the Nernst predicted value. Addition of the specific KCC2 blocker, VU0463271 (10 μM, 20 min), shifted E_*GABA*_ toward similar depolarized values in WT and *Mecp2*^–/*y*^ CA3 pyramidal neurons (*U* = 17.5, *P* = 0.5, Figures 5E,F). Altogether, these data indicate the presence of a functional, and indistinguishable from WT, chloride extrusion mechanism in *Mecp2*^–/*y*^ CA3 pyramidal neurons at symptomatic stages.

## Discussion

Altered KCC2 expression and impaired chloride homeostasis have been well documented in rodent models of RTT before the overt symptomatic period (Banerjee et al., 2016; Lozovaya et al., 2019; Scaramuzza et al., 2021). However, to the best of our knowledge, no study has been reporting a detailed evaluation of the strength of GABAergic inhibition at fully symptomatic stages. In the present study, we used early (P30–35) and late symptomatic (P50–60) male *Mecp2*-null mice, exhibiting the broad spectrum of phenotypes reported in RTT mouse models (Guy et al., 2001; Pratte et al., 2011; Calfa et al., 2015; Lozovaya et al., 2019) and found (i) no difference in the relative amount, but an over-phosphorylation, of KCC2 and NKCC1 between WT and *Mecp2*^–/*y*^ hippocampi and (ii) no difference in the inhibitory strength of GABA_*A*_-receptor activation nor in the reversal potential of evoked GABAergic synaptic currents between WT and *Mecp2*^–/*y*^ CA3 pyramidal neurons at any stages studied.

In the present study, the synaptic or agonist activation of GABA_*A*_ receptors hyperpolarizes (perforated patch-clamp recordings) and inhibits (loose patch-clamp and multi-unit activity recordings) WT and *Mecp2*^–/*y*^ CA3 pyramidal neurons with the same efficacy. A previous study reported that GABA_*A*_-receptor-mediated responses onto CA3 pyramidal neurons remain depolarizing and excitatory in presymptomatic (i.e., at birth and P15) male *Mecp2*^–/*y*^ mice (Lozovaya et al., 2019). In agreement with this finding, our perforated patch-clamp recordings showed that the evoked GABA_*A*_-receptor-mediated postsynaptic currents reversed polarity at a more depolarized potential in *Mecp2*^–/*y*^ CA3 pyramidal neurons compared to WT at P15 (data not shown). Combined with the result of the present work, these data indicate that, at least in the *Mecp2*^–/*y*^ CA3 pyramidal neurons, the GABAergic developmental sequence is delayed at pre-symptomatic stages and restored at symptomatic stages. Interestingly, a recent study reported that the expression of KCC2 is downregulated in cortices of presymptomatic but not symptomatic female *Mecp2*^–/+^ mice (Oyarzabal et al., 2020), further suggesting that a transient alteration of GABAergic inhibition takes place in this RTT mouse model. Disruptions of the GABAergic developmental sequence have been reported to alter the trajectory of brain development, hence functioning in adulthood. They are, for example, responsible for the excitatory/inhibitory imbalance in different mouse models of neurodevelopmental disorders (Kang et al., 2019; Pisella et al., 2019) including RTT (Lozovaya et al., 2019), and could therefore contribute to some of the RTT symptoms.

Tang et al. (2016) have shown that KCC2 is a target of Mecp2 and that compounds enhancing the expression of KCC2 rescued the morpho-functional abnormalities of human RTT neurons and exerted therapeutic effects in early symptomatic *Mecp2*^–/*y*^ mice (Tang et al., 2019). Based on our study, this therapeutic action may be accounted for by a rescue of chloride homeostasis in non-hippocampal brain regions or, alternatively, mediated through interactions with a wide range of cytoskeleton-associated and/or membrane proteins, independent of its Cl^–^ transport function (Virtanen et al., 2021), as reported for the regulatory role of KCC2 on spines formation (Li et al., 2007), synaptic plasticity (Gauvain et al., 2011; Chevy et al., 2015), and neuronal excitability (Goutierre et al., 2019). Examination of chloride homeostasis in additional brain regions at symptomatic stages would be interesting to address these points. However, it is worth noting that potentiating or activating the GABAergic system at the symptomatic stages led to several beneficial effects in both male and female *Mecp2*-deficient mice (Abdala et al., 2010; Voituron and Hilaire, 2011; El-Khoury et al., 2014), an observation not compatible with a permanent alteration of chloride homeostasis in *Mecp2*-deficient mice.

We did not observe any significant difference in the total amount of KCC2, but the phosphorylated forms were increased in the hippocampus of fully symptomatic *Mecp2*^–/*y*^ mice. Although previous studies demonstrated that threonine and serine residues regulate KCC2 function *in vivo* (Moore et al., 2019; Pisella et al., 2019), this over-phosphorylation of KCC2 does not induce any significant changes in the strength of GABAergic inhibition nor on the rate of chloride extrusion of *Mecp2*^–/*y*^ CA3 pyramidal neurons. One possible explanation for this discrepancy could rely on the regional and cellular heterogeneity present in the whole hippocampus, and we cannot exclude that the changes in the level of KCC2 phosphorylation, observed in the whole hippocampi, affect other neuronal populations than the one we recorded from. Alternatively, although the phosphorylation of KCC2 might affect its activity, the overall increase of KCC2 expression is sufficient to maintain normal chloride homeostasis in mature neurons as observed in transgenic mice carrying (de)phospho-mimetic mutations of KCC2 (Moore et al., 2019; Pisella et al., 2019).

To the best of our knowledge, the present study provides the first evidence of post-translational modifications of KCC2 and NKCC1 in neurological disease. Whether this over-phosphorylation is a cause or a consequence of the disease remains to be elucidated. However, recent studies using mouse models with mutations impairing the phosphorylation of KCC2 at serine 940 and threonine 906/1007 residues revealed that these mice exhibited altered seizures susceptibility (Silayeva et al., 2015; Moore et al., 2018) and symptomatic manifestations of neurodevelopmental disorders (Moore et al., 2019; Pisella et al., 2019). Of note, these mutations do not prevent the developmental switch from depolarizing to hyperpolarizing GABA, but rather alter the timing of this switch.

In summary, our study indicates the presence of a functional chloride extrusion mechanism in *Mecp2*^–/*y*^ CA3 pyramidal neurons at fully symptomatic stages. A previous study reported that chloride extrusion and KCC2 expression are decreased at presymptomatic stages, suggesting that the GABAergic developmental sequence is altered but not abolished in these neurons. Whether this observation applies to other hippocampal cell types and/or brain structures remains to be determined. This issue is crucial to maximize the development of chloride homeostasis-based therapeutic strategies.

## Data Availability Statement

The raw data supporting the conclusions of this article will be made available by the authors, without undue reservation.

## Ethics Statement

The animal study was reviewed and approved by the Ethical Committee for Animal Experimentation (APAFIS-Number 17605-2018-1119-1115-7094) delivered by the French Ministry of Education and Research.

## Author Contributions

J-LG, GW, and YB conceived the experiments and wrote the manuscript. J-LG, YB, DD, JZ, FB, and AM performed the experiments. J-CG bred the colony. All authors approved the final version of the manuscript.

## Conflict of Interest

The authors declare that the research was conducted in the absence of any commercial or financial relationships that could be construed as a potential conflict of interest.

## Publisher’s Note

All claims expressed in this article are solely those of the authors and do not necessarily represent those of their affiliated organizations, or those of the publisher, the editors and the reviewers. Any product that may be evaluated in this article, or claim that may be made by its manufacturer, is not guaranteed or endorsed by the publisher.
